# Alterations in pro- and anti-inflammatory mediators are involved in microvascular dysfunction in postmenopausal women with type 2 diabetes mellitus

**DOI:** 10.1590/1414-431X2021e11821

**Published:** 2022-02-28

**Authors:** A.P. Jarrete, L.T. Giollo-Junior, J.F. Vilela-Martin, I.P. Novais, M.A. Delbin, A. Zanesco

**Affiliations:** 1 Departamento de Biologia Estrutural e Funcional, Universidade Estadual de Campinas, Campinas, SP, Brasil; 2 Posto Médico Garrison - 5^a^ Brigada de Cavalaria Blindada, Exército Brasileiro, Ponta Grossa, PR, Brasil; 3 Departamento de Medicina, Faculdade de Medicina de São José do Rio Preto, São José do Rio Preto, SP, Brasil; 4 Departamento de Saúde I, Universidade Estadual do Sudoeste da Bahia, Jequié, BA, Brasil; 5 Programa de Pós-Graduação em Saúde e Meio-ambiente, Faculdade de Medicina, Universidade Metropolitana de Santos, Santos, SP, Brasil

**Keywords:** Type 2 diabetes, Microvascular function, Postmenopausal women, Sex differences, Circulatory biomarkers

## Abstract

Evidence has shown that women with type 2 diabetes mellitus (T2DM) have a greater risk of cardiovascular complications compared with men, but this sex difference is not clearly understood. This study assessed the microvascular function and circulatory biomarkers in postmenopausal women (PMW) with T2DM compared with diabetic men and their non-diabetic counterparts. Sixty participants were divided into nondiabetic PMW, PMW with T2DM, non-diabetic men, and diabetic men. Microvascular function was assessed using non-invasive equipment (EndoPAT^®^) and reported as reactive hyperemia index (RHI). Anthropometric and cardiovascular parameters were also measured. Two-way ANOVA was performed using sex (women or men) and T2DM (non-diabetic and diabetic) as the two factors. RHI impairment (1.97±0.14) was detected in diabetic PMW compared with women without T2DM (2.5±0.13) accompanied by lower adiponectin levels (T2DM: 9.3±1.2 and CTL: 13.8±1.8 ug/mL, P<0.05). An increase in the Nε-carboxymethyllysine (CML), nitrate/nitrite, and C-reactive protein (CRP) levels were observed in diabetic PMW compared to the other groups. Although a poor glycemia control was seen in diabetic men, neither RHI nor circulatory biomarkers were affected by T2DM. Multiple linear regression stratified by sex and T2DM identified some variables with RHI only in PMW with T2DM: HbA1c (P=0.003), body mass index (P=0.029), CML (P=0.032), and CRP (P=0.006). Diabetic PMW were more susceptible to the deleterious effects of hyperglycemia than men, showing microvascular dysfunction with high levels of pro-inflammatory mediators (CML and CRP) and a lower adiponectin concentration.

## Introduction

Cardiovascular complications are common events in patients with type 2 diabetes mellitus (T2DM) with high morbidity and mortality, with a 2- to 3-fold increased risk of stroke, myocardial infarction, and peripheral artery disease compared with nondiabetic individuals (1). It has been shown that women with T2DM are at greater risk of cardiovascular diseases (CVD) than men by 4-5-fold (2-4). Nevertheless, this positive interaction between T2DM and CVD in women is not conclusive. Women with diabetes have a 44% higher risk of myocardial infarction compared to men with diabetes (5-7), whereas other studies showed that rates of a coronary artery disease event were similar among women and men with T2DM (8), and elevated glycated hemoglobin (HbA1c) had a similar adverse effect on myocardial infarction in women and men (9). Therefore, studies that evaluate vascular function associated with diabetes biomarkers may clarify sex differences and help the health care system consider this issue and pay special attention to diabetic women in the prevention of cardiovascular complications.

Another issue related to sex differences in the diabetic status is to identify which risk factor(s) is/are more likely to be associated with CVD in women with T2DM compared with men. Previous studies have shown that women are more sensitive to risk factors for CVD than men, such as body mass index (BMI), abdominal circumference, total cholesterol, triglycerides, and low-density lipoprotein (LDL)-cholesterol (10,11). Additionally, evidence has pointed out the importance of pro- and anti-inflammatory mediators, sex hormones, and endothelial dysfunction on CVD in diabetic women (12). Given that women are more susceptible to CVD after menopause regardless of preexisting conditions (13), assessment of circulatory biomarkers associated with microvascular function in postmenopausal women with T2DM could contribute to prevention strategies for women's cardiovascular health. In addition, women live longer than men, making prevention (primary or secondary) a priority in women's health care, since healthy aging is essential for maintaining functional ability and being economically active in old age (14).

Therefore, this study aimed at: 1) assessing whether T2DM affects microvascular function equally in postmenopausal women and men at a similar age compared with their nondiabetic counterparts; and 2) investigating circulatory biomarkers directly related to: a) T2DM (glycemia, HbA1c, Nε-carboxymethyllysine (CML), and advanced glycation end products (AGEs)); b) CVD (nitrate/nitrite, C-reactive protein (CRP), and lipid profile); and c) pro- and anti-inflammatory adipokines, tumor necrosis factor-alpha (TNF-alpha), and adiponectin.

## Material and Methods

### Participants

The study was approved by the Ethics Committee of the University of Campinas (UNICAMP-CAAE: 53104516.3.0000.5404) following the principles outlined in the Declaration of Helsinki and the STROBE cross-sectional reporting guidelines (15). The participants were divided into four groups: nondiabetic postmenopausal women (control, n=16), postmenopausal women with T2DM (n=15), nondiabetic men (control, n=15), and men with T2DM (n=14). The postmenopausal period was determined as the absence of menstruation for at least one year due to natural or surgical causes. T2DM was determined based on a previous medical diagnosis. The inclusion criteria were that women and men be physically inactive and a similar time after menopause for women. The exclusion criteria were smokers, taking hormone replacement, history of cardiovascular events, autoimmune diseases, renal dysfunction, and insulin therapy. According to these criteria, 125 participants were enrolled between June 2016 and November 2018, but only sixty were eligible for the study. Before starting the protocol, the participants were informed about the procedures and risks of the study and signed a consent form in accordance with UNICAMP's Ethics Committee.

### Study design

All groups underwent the same procedures (between 7:00 and 10:00 a.m.), following a predetermined order. In the first appointment, the medical records were assessed and the medication inventory, anthropometric parameters, resting heart rate (HR), systolic blood pressure (SBP), diastolic blood pressure (DBP), and microvascular function were measured. In the second appointment (scheduled within 7 days after the first appointment), blood samples were collected after a 12-h fast, with participants being instructed to avoid physical activities, not to take vitamins, supplements, and caffeine and, if possible, avoid using certain medications such as β-blockers, nitrates, calcium channel blockers, anti-inflammatories, and aspirin at least 24 h before the evaluations.

### Anthropometric and cardiovascular measurements

Body weight and height were measured, BMI was calculated, and the waist circumference (WC) was taken as described previously (16). After 20 min of seated rest, three consecutive BP measurements were taken using semiautomatic equipment with oscillometric devices calibrated periodically (Microlife, MIB-P3BTOA, Brazil). Resting BP was determined as the average of three measurements. The resting BP values were included in the information about participants required by the EndoPAT^®^ software before starting the microvascular function test. Hypertensive participants were classified according to previous medical diagnoses.

### Microvascular function test

Microvascular function was performed in a quiet and temperature-controlled (24-27°C) room and obtained using non-invasive equipment (EndoPAT 2000, Itamar Medical Inc., Israel) as previously described (17). This technique evaluates beat-by-beat plethysmographic measurements of pulse wave amplitude in the fingers through probes. Briefly, the protocol included a 10-min baseline record, followed by 5-min inflation of a blood pressure cuff around the non-dominant arm (tested arm) with a pressure of 60 mmHg above resting SBP. Finally, a 5-min signal was recorded after deflation of the cuff, representing hyperemic response. Blood pressure cuff occlusion was not applied to the contralateral arm (control arm). The primary output of the EndoPAT examination is the reactive hyperemia index (RHI), representing the microvascular function. RHI is automatically calculated through a computer algorithm by normalizing the baseline signal and indexing the ratio on the test arm to that of the control arm, following the equation: [(tested arm-post occlusion / tested arm-baseline) / (control arm-post occlusion / control arm-baseline)] × baseline correction factor. As a secondary output of this device, the mean resting HR was also evaluated concomitantly during the baseline period.

### Biochemical analyses

Blood samples were centrifuged at 1,000 *g* for 15 min at room temperature, and the plasma and serum were stored at -80°C for biochemical analyses. Glycated hemoglobin (HbA1c), glycemia, serum lipid profile, estradiol, and creatinine were determined using an automated standard method (Fleury Laboratory, Brazil). Plasma adiponectin (Millipore, USA, catalogue number: EZHADP-61K), serum C-reactive protein (Cayman Chemical, USA, catalogue number: 10011236), plasma nitrate/nitrite (NOx^-^) (Cayman Chemical, catalogue number: 780001), serum Nε-carboxymethyllysine (Cell Biolabs, USA, catalogue number: STA-816), and serum tumor necrosis factor α (Millipore, catalogue number: HCYTOMAG-60K) were measured using a commercial kit according to manufacturers’ instructions. The glomerular filtration rate (GFR) was determined according to the equation (18):

$$\begin{array}{c} {\text{C}}_{\text{Cr}}=\left\{\left(140-\text{age}\right)\times \text{weight/}\left(72\times {\text{S}}_{\text{Cr}}\right)\right\}\times 0.85\\ \left(\text{if female}\right) \end{array}$$ (Eq. 1)

### Statistical analysis

Data are reported as means±SE. The normality and homogeneity of the data were verified by the Kolmogorov-Smirnov and Levene tests, respectively. The variables that did not present a normal distribution were standardized using a z-score. Two-way ANOVA was performed using sex (women or men) and T2DM (non-diabetic and diabetic) as the two factors followed by Bonferroni's *post hoc* test for comparing the differences between groups. A multiple linear regression model was performed: 1) the RHI was established as the dependent variable and sex and T2DM as the independent variables. Secondary analyses were stratified by sex and T2DM; 2) the RHI was established as the dependent variable and the HbA1c, BMI, CML, adiponectin, and CRP levels as the independent variables (IBM SPSS 23.0, SPSS Inc., USA). The power of the study was 95% for all analyzed groups. P<0.05 was considered statistically significant. All figures were constructed using GraphPad Software (Prism 5, USA).

## Results

Higher WC, BMI, and GFR values were found in women with T2DM while total cholesterol, LDL-C, and non-high-density lipoprotein (HDL)-C concentrations were lower compared with non-diabetic women (P<0.05). A lower level in LDL-C concentration was found in men with T2DM compared with non-diabetic men (P<0.05). No differences were observed in HDL-C and triglycerides levels in all groups (Table 1). The concentration of creatinine and systolic and diastolic blood pressure values were higher in both diabetic and non-diabetic men compared to their respective women's counterpart group (P<0.05), even though the values were within a normal range. Table 1 summarizes the data.

**Table 1:** Characteristics of the study participants.

	Women		Men	
	Control (n=16)	T2DM (n=15)	Control (n=15)	T2DM (n=14)
Age (years)	56.7±0.8	57.7±1.0	54.2±1.3	55.8±1.6
BMI (kg/m^2^)	27.2±0.8	30.7±0.8*	29.1±1.2	29.8±1.0
WC (cm)	92.0±2.5	101.9±2.2*	100.5±2.4^†^	105.4±2.7
Postmenopausal time (y)	8.1±1.4	9.0±1.1	-	-
Estradiol (ng/mL)	0.88±0.17	0.66±0.08	-	-
Fasting insulin (mU/L)	9.5±0.6	14.6±3.3	13.3±0.8	17.4±3.9
Creatinine (mg/dL)	0.71±0.03	0.72±0.04	1.02±0.04^†^	1.04±0.12^‡^
GFR (mL/min)	94.5±3.7	113.9±6.2*	100.3±5.1	110.5±8.6
SBP (mmHg)	115.8±2.8	121.4±2.7	123.8±4.1	130.7±2.8^‡^
DBP (mmHg)	73.8±2.3	76.5±2.1	81.6±1.9^†^	82.9±1.8^‡^
Rest heart rate (bpm)	65.1±1.9	64.9±1.9	66.0±3.1	65.0±2.4
Total cholesterol (mg/dL)	214.9±8.8	186.2±6.0*	191.5±7.3^†^	174.4±7.6
LDL-C (mg/dL)	133.9±6.6	104.9±6.0*	118.7±7.0	96.4±7.1^#^
HDL-C (mg/dL)	51.7±4.0	53.2±2.9	44.9±2.0	46.5±3.6
Non-HDL (mg/mL)	163.2±8.6	133.0±6.3*	146.5±7.7	125.9±9.4
Triglycerides (mg/dL)	162.5±19.0	154.7±15.1	153.1±18.3	191.1±39.7

BMI: body mass index; WC: waist circumference; GFR: glomerular filtration rate; SBP: systolic blood pressure; DBP: diastolic blood pressure; LDL-C: low-density lipoprotein cholesterol; HDL-C: high-density lipoprotein cholesterol. Data are reported as means±SE. *P<0.05 *vs* control women; ^#^P<0.05 *vs* control men; ^†^P<0.05 *vs* control women; ^‡^P<0.05 *vs* type-2 diabetes (T2DM) women (two-way ANOVA).

All the participants with T2DM were on oral hypoglycemic medication. Regarding oral antidiabetic medication, most of the women with T2DM were taking biguanides (86%), followed by sulphonylureas (40%), dipeptidyl peptidase 4 inhibitors (DPP-4, 34%), sodium-glucose cotransporter (SGLT2, 13.4%), and thiazolidinedione (TZD, 7%). Similarly, biguanides were the major class of oral antidiabetic treatment in men with T2DM (86%), followed by sulphonylureas (50%) and DPP-4 inhibitors (21%). Approximately 54 and 64% of women and men with T2DM were hypertensive, respectively.

### Circulatory biomarkers and microvascular function

**Figure 1:**
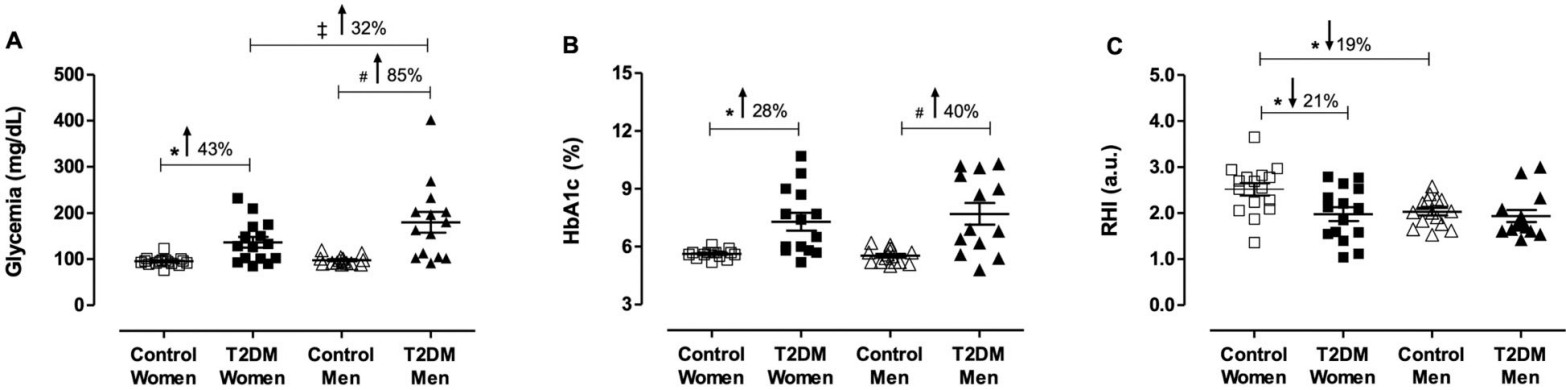
Microvascular function and glycemic profile of groups. Groups: non-diabetic postmenopausal women (control women), postmenopausal women with type-2 diabetes (T2DM women), non-diabetic men (control men), and men with T2DM (T2DM men). **A**, Glycemia; **B**, Glycated hemoglobin A1c (HbA1c); **C**, Reactive hyperemia index (RHI, a.u. arbitrary unit). Data are reported as means±SE. *^#‡^P<0.05 (two-way ANOVA).

To examine the circulatory biomarkers in T2DM groups and to detect the differences between the sexes, we evaluated biomarkers directly related to this disorder and their associations with microvascular function. As expected, participants with T2DM presented higher glycemia (43 and 85% for women and men, respectively) and HbA1c values (28 and 40% for women and men, respectively) compared to their nondiabetic counterparts (Figure 1A and B). A striking sex difference was found between the two diabetic groups regarding glycemia, with a worse glycemic control in men, approximately 32% (P<0.05) (Figure 1A). Surprisingly, women with T2DM had significant impairment in vascular function (P<0.05) measured indirectly by RHI, approximately 21% (control: 2.51±0.13 *vs* T2DM: 1.97±0.14), while diabetes did not affect RHI in men (control: 2.05±0.08 and T2DM: 2.00±0.13) (Figure 1C). Another difference between the sexes was found when comparing RHI in the control groups; healthy women had a greater RHI (2.51±0.13) compared to men (2.00±0.13), approximately 19%, (P<0.05) (Figure 1C).

**Figure 2:**
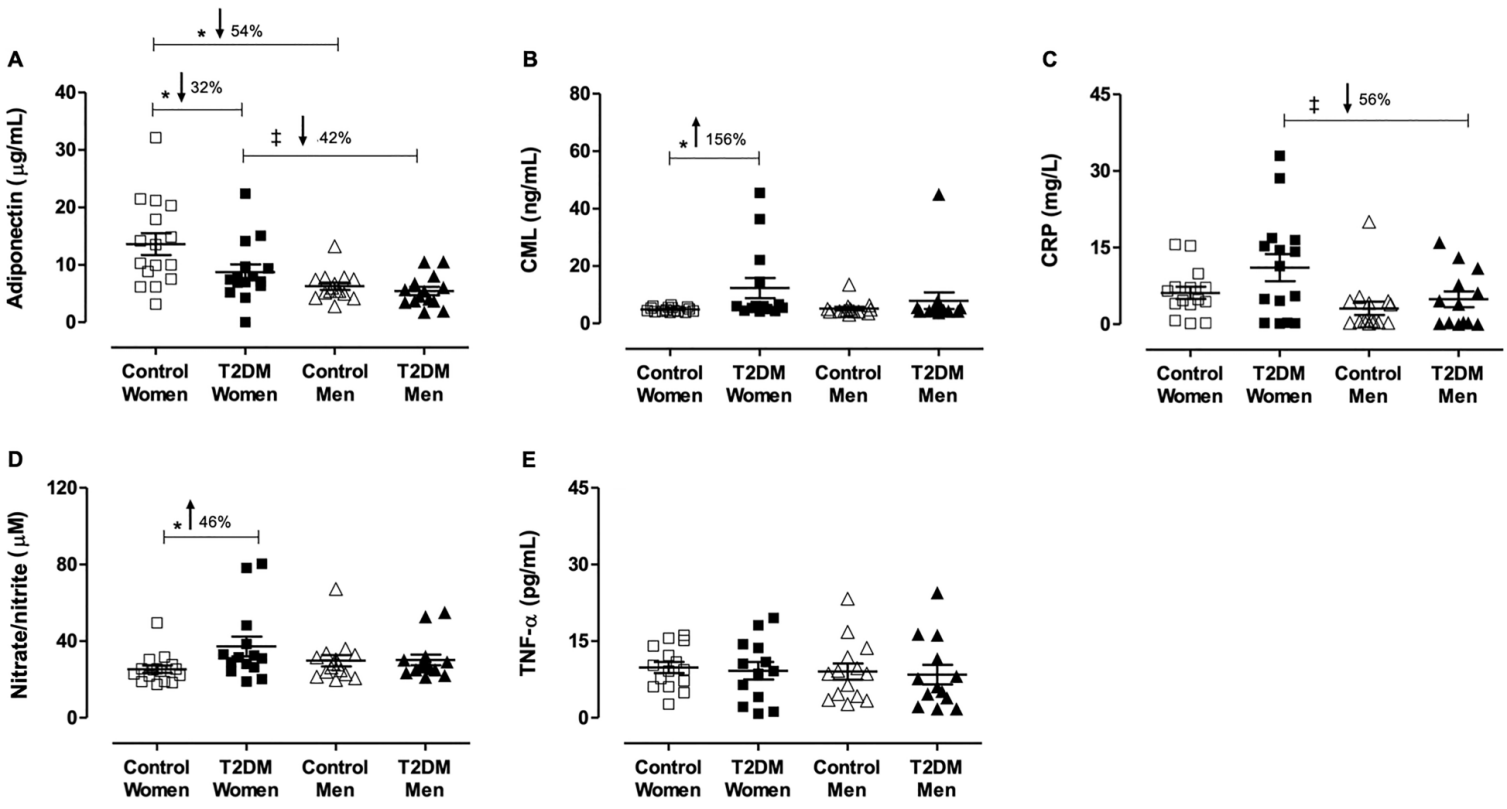
Circulatory biomarkers. Groups: nondiabetic postmenopausal women (control women), postmenopausal women with type-2 diabetes (T2DM women), non-diabetic men (control men), and men with T2DM (T2DM men). **A**, Adiponectin; **B**, Nε-carboxymethyllysine (CML); **C**, C-reactive protein (CRP); **D**, Nitrate/nitrite; **E**, Tumor necrosis factor-alpha (TNF-α). Data are reported as means±SE. *^‡^P<0.05 (two-way ANOVA).

Figure 2 shows the circulatory anti- and pro-inflammatory mediators in all studied groups. The presence of T2DM significantly affected the concentration of adiponectin in postmenopausal women, about 32% of reduction (control: 13.8±1.8 and T2DM women: 9.3±1.2 ug/mL, P<0.05), whereas this anti-inflammatory adipokine was not altered in men (control men: 6.2±0.6 *vs* T2DM men: 5.4±0.7 ug/mL, P>0.05). As expected, a sex difference was observed in adiponectin level both in nondiabetic and diabetic groups (Figure 2A).

The concentrations of CML were markedly higher in postmenopausal women with T2DM (P<0.05), approximately 156% compared with their non-diabetic counterparts (Figure 2B). Additionally, CRP levels were markedly higher (56%) in women with T2DM compared with diabetic men (Figure 2C). Overall, diabetic status did not affect the levels of these pro-inflammatory mediators in men (Figure 2B and C). Confirming the sex difference in the susceptibility of the pro-inflammatory response, the NOx_-_ levels were also increased in diabetic postmenopausal women, approximately 46% (Figure 2D). The TNF-α levels were not different between the groups (Figure 2E).

The multiple linear regression model identified the independent variables for RHI: sex (P=0.009) and T2DM (P=0.018), data not shown. In addition, the multiple linear regression stratified by sex and T2DM identified some variables with RHI only in women with T2DM: HbA1c (P=0.003), BMI (P=0.029), CML (P=0.032), and CRP (P=0.006) (Table 2).

**Table 2:** Multiple linear regression for reactive hyperemia index (RHI) and related variables.

Variables	Control			T2DM		
	Coefficients	95%CI	P-value	Coefficients	95%CI	P-value
Women						
HbA1c	0.523±0.730	-1.160 to 2.206	0.494	-0.332±0.075	-0.510 to -0.154	0.003
BMI	-0.017±0.057	-0.149 to 0.115	0.770	-0.085±0.031	-0.159 to -0.012	0.029
CML	0.101±0.249	-0.473 to 0.674	0.697	0.031±0.012	0.004 to 0.059	0.032
Adiponectin	-0.001±0.022	-0.051 to 0.049	0.957	0.020±0.026	-0.041 to 0.082	0.460
CRP	-0.032±0.040	0.123 to 0.059	0.439	0.054±0.014	0.021 to 0.087	0.006
Intercept	-0.179±3.821	-8.992 to 8.633	0.964	5.974±1.030	3.539 to 8.409	0.001
Men						
HbA1c	0.143±0.300	-0.536 to -0.822	0.646	-0.091±0.080	-0.280 to 0.098	0.291
BMI	0.006±0.021	-0.041 to 0.052	0.793	0.127±0.089	-0.084 to 0.338	0.198
CML	0.016±0.040	-0.075 to 0.108	0.700	-0.044±0.026	-0.106 to 0.017	0.133
Adiponectin	-0.015±0.045	-0.117 to 0.087	0.748	0.062±0.064	-0.088 to 0.213	0.360
CRP	0.021±0.021	-0.068 to 0.026	0.333	-0.034±0.029	-0.102 to 0.033	0.271
Intercept	1.157±1.890	-3.119 to 5.433	0.556	-1.039±2.610	-7.210 to 5.133	0.703

HbA1c: glycated hemoglobin A1c; BMI: body mass index; CML: Nε-carboxymethyllysine; CRP: C-reactive protein.

## Discussion

The main finding of this study was that postmenopausal women with T2DM without previous CVD events had impaired microvascular function that was accompanied by a high plasma concentration of two pro-inflammatory mediators, CML and CRP, and a lower level of anti-inflammatory adipokine, adiponectin, even though glycemia was significantly lower compared to diabetic men at a similar age.

It has been systematically shown that several pathways explain the cardiovascular complications associated with T2DM in the general population (7,12,19), but no studies have examined the impact of T2DM on microvascular function in postmenopausal women comparing them to nondiabetic counterparts and males. In addition, anthropometric parameters, BP, GFR, and lipid profile are considered important risk factors for cardiovascular complications for both men and women with T2DM (10). WC was significantly higher in diabetic women (10%) even though this anthropometric parameter was above the normal values in the female groups, according to the WHO guidelines (14). Indeed, it is well known that women after menopause show a significant change in anthropometric parameters because of sex hormone deficiencies (20). Additionally, fat distribution and metabolic rate differ between men and women throughout life, depending on genetics, the amount of skeletal muscle mass, and the density of adrenergic receptor subtypes in fat depots (20). Given that GFR is considered a gold standard indicator of renal function, we measured this parameter in all groups to exclude interfering factors in the circulating biomarkers of interest. Although postmenopausal women with T2DM showed higher values for GFR compared with their non-diabetic counterparts, the values were still within normal range, so this parameter can be excluded as a confounding factor (21).

Previous studies have demonstrated a pathological link between obesity, lower adiponectin level, and insulin resistance in both men and women (22-24). Conversely, a study showed no association between trunk fat and adiponectin level in middle-aged and older women with or without diabetes (25). Accordingly, we found no correlation between WC and the anti-inflammatory mediator, adiponectin, in postmenopausal women with T2DM (P=0.16, data not shown). The multiple linear regression indicated a statistical relationship between BMI and RHI in women with T2DM, and a significant decrease in the concentration of adiponectin was detected in diabetic women. Accordingly, a population-based cohort examining 2,400 men and women reported that high adiponectin levels is associated with a lower incidence of T2DM, and this association is stronger in women than in men (26). Adiponectin also plays an antiatherogenic role in modulating macrophage to foam cell transformation preventing vascular damage during the atherogenesis process (27). Taken together, the lower concentration of adiponectin observed in postmenopausal diabetic women may contribute to an impairment of vascular function in this population. It should also be emphasized that the sex differences between the groups have already been published by our group, where women had higher adiponectin levels compared to men in a healthy population (28).

Chronic inflammation, and subsequent oxidative stress, plays a key role in diabetic status and its complications, which are characterized by a marked increase in pro-inflammatory mediators such as interleukin (IL)-1β, IL-6, TNF-α, CRP, and other IL-1β dependent cytokines (19). Moreover, the activation of the AGEs/RAGE/NFkB pathway is strongly associated with cardiovascular complications in both types of DM (29-31). The AGEs are a group of heterogeneous substances, and CML is one of the most studied among them. Our findings clearly showed that the pro-inflammatory mediators CRP and CML were markedly elevated in postmenopausal women with T2DM compared with non-diabetic counterparts and diabetic and non-diabetic men. It is intriguing that no differences were found in TNF-α levels in all studied groups. Collectively, our findings showed that postmenopausal women were more susceptible to diabetic status with significant alterations in pro- and anti-inflammatory mediators that are accompanied by microvascular dysfunction even though glycemia and HbA1c were lower than those in men. These results revealed an important clinical characteristic; in addition to the classical glucose regulation, the biomarkers AGEs, CRP, and adiponectin are good predictors of vascular function in postmenopausal women. Accordingly, a previous study has shown that the early detection of AGEs is an important prognostic factor for diabetic nephropathies, and increased serum levels of CML in diabetic subjects indicated ongoing glycemia damage and their susceptibility to developing renal complications (19). Most studies involving AGEs and vascular function have been carried out in experimental diabetes models (32) or isolated cells (33). Our study is the first to examine this important biomarker directly involved in the CVD complication in diabetic postmenopausal women and the differences between the sexes. Another point that should be addressed is the lack of changes in vascular function in diabetic men, as well as the circulatory pro- and anti-inflammatory biomarkers. An early study has demonstrated that CML is associated with an increased risk of coronary heart disease in older populations, but they examined women and men altogether, and 64.4% of the studied population were women (34). Conversely, when men were studied separately, there were no significant differences in the concentration of AGEs, including CML, in older men with (n=455) or without diabetes (n=2,566) even though the fasting glycemia was higher in patients with diabetes (35). Accordingly, our study clearly shows that the concentration of CML is not affected by diabetes in men, but it is in women.

Regarding the NOx^-^ level, results were controversial. Data from our laboratory showed that in experimental diabetes models that used male rats, a significant reduction in nitric oxide (NO) bioavailability takes place (36), whereas this parameter showed great variability in human studies, ranging from 10±0.9 up to 25±1.4 µM (16,35). In the present study, we found an increase in NOx_-_ level (46%) in postmenopausal women with T2DM, indicating an overproduction of NO that could reflect an inflammatory condition in this population. Accordingly, a meta-analysis found that both T1DM and T2DM participants showed an increased level of NOx_-_ compared with the control group with up-regulation of inducible NO synthase (iNOS) and a massive generation of NO in response to inflammation and oxidative stress (37).

A limitation of this study was the small number of participants. Nevertheless, our study focused on a narrow age bracket since a significant disparity between the early and late postmenopausal periods has been reported, affecting data integrity. Thus, the narrowness of the inclusion/exclusion criteria was important to get a homogenous population.

### Conclusion

**Figure 3:**
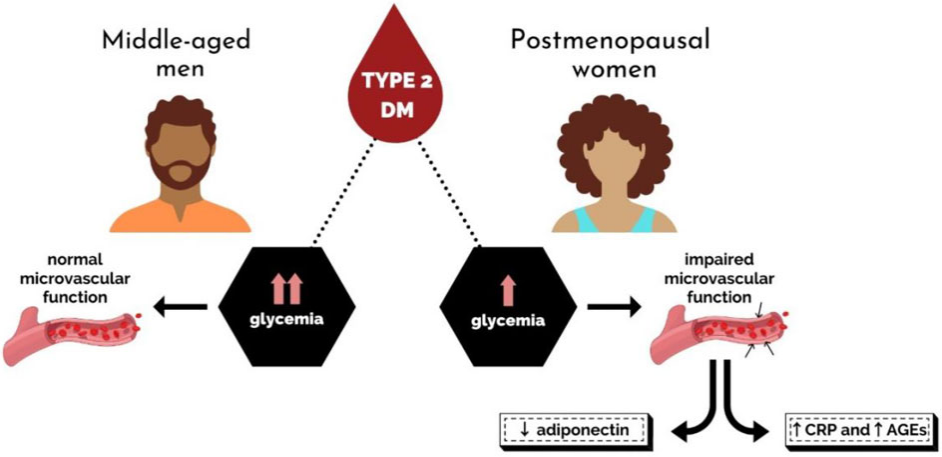
Graphical abstract showing that vascular dysfunction is associated with lower production of adiponectin and overproduction of the pro-inflammatory mediators advanced glycation end products (AGEs) and C-reactive protein (CRP) in diabetic postmenopausal women, even though glycemia was lower than in men.

Our findings showed that vascular dysfunction was associated with two pathways, lower production of the anti-inflammatory agent adiponectin and overproduction of the pro-inflammatory mediators AGEs and CRP in diabetic postmenopausal women, even though the classical biomarker for T2DM (glycemia) was lower than in men. A graphical abstract illustrates this conclusion (Figure 3).
